# Triptolide suppresses melanoma cell growth *in vitro* and *in vivo* through the Src-ERK signaling pathway

**DOI:** 10.7150/jca.100840

**Published:** 2024-10-14

**Authors:** Haibo Zhang, Zhiqiang Zhang, Miao Jiang, Shengchao Wang, Jing Wang, Hui Wang, Yanjie Liu, Youxu Wang, Junmin Fu, Penglei Wang, Mingsan Miao, Myoung Ok Kim, Xiaoyan Fang

**Affiliations:** 1 College of pharmacy, Henan University of Chinese Medicine, Zhengzhou 450045, Henan Province, China.; 2 Collaborative Innovation Center of Research and Development on the Whole Industry Chain of Yu-Yao, Henan Province, China.; 3 First Affiliated Hospital of Henan University of Chinese Medicine, Henan Children's Hospital of Integrated Traditional Chinese and Western Medicine, Zhengzhou 450004, China.; 4 Department of Animal Science and Biotechnology, Kyungpook National University, Sangju-si, Republic of Korea.

**Keywords:** triptolide, melanoma, Src-ERK pathway, proliferation, metastasis

## Abstract

Melanoma is a highly aggressive form of skin cancer with a rapidly increasing incidence. New strategies are urgently needed for treating advanced melanoma which is closely linked to metastasis and often results in death. The Src-ERK signaling axis contributes significantly to both cell growth and metastasis. Triptolide, a *Tripterygium wilfordii* extract used for treating autoimmune conditions in traditional Chinese medicine, also has anti-inflammatory, neuroprotective, and antitumor activities. However, its ability to treat melanoma, including its target and underlying mechanism, requires clarification. We performed a range of *in vivo* and *in vitro* cellular experiments, encompassing assessments of cell proliferation, cell cycle progression, apoptosis, migration, and invasion, alongside nude mouse xenograft tumor studies, to evaluate the therapeutic potential of triptolide in melanoma. Here, it was found that triptolide markedly reduced proliferation, invasion, and migration in SK-MEL-5 and SK-MEL-28 cells. Triptolide was shown to arrest the cell cycle in G0/G1 and induce apoptosis, with further investigation showing that these effects were mediated by the Src-ERK pathway. Thus, the findings indicated that triptolide could inhibit melanoma cell growth and metastasis, suggesting its potential for treating metastatic melanoma.

## Introduction

Melanoma originates in melanocytes in the epidermis, mucosa, and other tissues. Annual statistics estimate approximately 280 000 new diagnoses and over 60 000 deaths globally 1. The tumor is associated with both high mortality and unfavorable prognosis 2, 3. Surgery remains the main option, although new treatments, such as immunotherapy, have been used 4-6. However, these new treatments are only effective for some patients. Chemotherapeutic agents such as paclitaxel, cisplatin, temozolomide, and dacarbazine have various issues, including poor efficacy and significant adverse reactions. Various signaling pathways linked to tumorigenesis and metastasis have been found to be involved in melanoma 7.

The levels of Src family kinases (SFKs) are frequently elevated in many human cancers. This commonly results from upstream aberrant activation of growth factor-associated receptor tyrosine kinases. Src is a cytoplasmic tyrosine kinase that on activation, is transferred to the nucleus where it influences the activities of transcription factors, altering gene transcription 8. Aberrant stimulation of the MAPK/ERK axis promotes melanoma tumorigenesis. Targeting of ERK has thus been proposed for treating melanoma 9.

Triptolide, extracted from *Tripterygium wilfordii* Hook.f., is used as a traditional Chinese formulation for autoimmune conditions. It has recently also been found to have anticancer activities in tumor cells by activating caspases and p53 10. Triptolide has been found to augment apoptosis induced by carboplatin in melanoma 11, promote both apoptosis and autophagy in cutaneous squamous cell carcinoma 12, and inhibit cholangiocarcinoma proliferation in the liver through suppression of glycolysis 13 by the Akt/mTOR axis. It has also been shown to reduce the growth of gastric cancer cells by modulation of H19/NF-κB/FLIP signaling 14, as well as esophageal squamous cell cancer via the MAPK/ERK axis 15 and growth of B16F10 mouse melanoma cells via NF-κB 16. However, despite these findings, the mechanism underlying triptolide inhibition of melanoma growth has not been fully elucidated.

Here, the impact of triptolide on melanoma was investigated in both cells and mouse models, as well as its impact on the Src-ERK pathway, finding that triptolide inhibits melanoma cell growth through suppression of the Src-ERK axis.

## Materials and Methods

### Reagents and antibodies

Triptolide was bought from Sigma (USA) and was dissolved in DMSO to various working dilutions. Antibodies against Src (36D10), ERK1/2, p-ERK1/2 (Thr202/Tyr204), p53, cleaved caspase 3, Bax, and cyclin D1 were from Cell Signaling Technology (USA), while anti-p-Src (Tyr416) (GTX24816) was from GeneTex (Irvine, CA, USA) and anti-β-actin was from Santa Cruz Biotechnology (USA).

### Cell culture

Human melanoma SK-MEL5 and SK-MEL-28 cells, as well as human keratinocyte HaCaT cells, were acquired from the ATCC. Cells were grown in MEM or DMEM with 100 μg/mL streptomycin, 100 U/mL penicillin, and 10% fetal bovine serum (FBS; Gibco) at 37°C with 5% CO_2_ and 95% humidity.

### Cell viability

Viability was evaluated using CCK-8 assays (Dojindo Laboratories, Japan). Melanoma cells (2×10^3^/well) were grown in 96-well plates for 24 h before the addition of varying amounts of triptolide in DMSO for 0, 24, 48, and 72 h. The CCK-8 reagent (10 µL) was then added for 1 h at 37°C. The absorbance at 450 nm was determined with a microplate reader (BioTek, USA), using the mean of triplicate samples as the final reading.

### Assessment of anchorage-independent growth

Cells (8×10^3^/well) in complete medium with 0.3% agar and varying doses of triptolide or DMSO were layered on a layer of 0.5% agar, with both layers containing triptolide or vehicle as appropriate. After incubation for 2 weeks under normal growth conditions, the colonies were evaluated microscopically and counted using ImageJ software.

### Cell cycle and apoptosis

Cells (2.0 × 10^5^/dish) were inoculated in 60-mm dishes and incubated with varying doses of triptolide (0, 10, 20, and 40 nM) for 24 h. For cell cycle assessments, cells were fixed (70% ethanol, 16 h, -20℃), rinsed with pre-chilled PBS, and suspended in 250 μL of 0.6% Triton X-100. After incubation with RNaseA (200 μg/mL, 1 h, room temperature), cells were examined on a BD FACS Calibur flow cytometer (USA). To examine apoptosis, cells were stained with annexin V and propidium iodide (PI, 10 μg/mL) before flow cytometric analysis.

### Migration and invasion

Migration was evaluated with 24-well Transwell plates (pore size, 8 µm; Corning) following the provided directions. Cells (8×10^4^/100 µL serum-free DMEM) were inoculated with varying doses of triptolide in the upper chambers, while the lower chambers contained 600 μL of complete medium. After 48 h, cells were fixed (4% paraformaldehyde, 20 min) and stained with crystal violet (0.05%), while non-migrated cells were collected with cotton swabs. The same assay was used to assess invasion, although Matrigel was used to pre-coat the chamber. The numbers of invading cells were counted microscopically after staining.

### Immunofluorescence staining

SK-MEL-5 cells (1 × 10^5^/well) were plated on Lab-Tek II Chamber Slides (Thermo Fisher) and exposed to triptolide for 24 h, after which they were fixed (4% formaldehyde, 15 min), permeabilized (0.3% Triton X-100), blocked (5% BSA in PBS, 1 h), and treated with an anti-p-Src (Tyr416) antibody (1:50; overnight, 4°C). After exposure to a secondary antibody coupled to Alexa Fluor 488 (Jackson Laboratories, USA) at room temperature for 2 h, the coverslips were rinsed and mounted in fluorescent mounting medium containing DAPI, followed by evaluation and imaging under fluorescence microscopy (Leica).

### Western blotting

Cells were lysed, and centrifuged (12 000 rpm, 20 min), and protein contents were assessed using NanoDrop 2000 spectrophotomer (Thermo Fisher). Proteins were electrophoresed on SDS-PAGE and electroblotted to PVDF. Following blocking (5% BSA, 1 h), the blots were treated overnight (4 °C) with primary antibodies, followed by probing with appropriate secondary antibodies; washing between incubations was done with 1×TBST. Band visualization was performed using ECL (GE Healthcare) with quantification by Da Vinci software against β-actin.

### Xenograft mouse model

BALB/c nude mice (female, six weeks old) were acquired from Charles River and were kept for one week with a 12/12-h light/dark cycle before the experiment. The protocol received ethical approval from the Henan University of Chinese Medicine (DWLLGZR202202198). SK-MEL-5 cells (5×10^6^ in 200 μL PBS) were subcutaneously administered to 24 mice in the right rear flank. When the tumors had grown to approximately 100 mm^3^ in size, the animals were randomly allocated to three groups, with eight animals per group. One group was treated with vehicle only and the remaining two with 150 and 300 μg/kg, respectively. Injections were given intraperitoneally on alternate days for four weeks. The body weights were assessed twice a week with volumes evaluated as length × width × depth × 0.52. After the euthanasia of the mice, parts of the tumors were frozen in liquid nitrogen, and parts were fixed in 10% formalin.

### Immunohistochemical staining

The paraffin-embedded tumors were sectioned and subjected to antigen retrieval before blocking with 5% BSA and treatment at 4°C with primary antibodies against Ki-67 (1:100), p-Src (1:50), and p-ERK1/2 (1:100). Sections were probed with a secondary antibody, visualized with DAB, and counterstained with hematoxylin before imaging and evaluation with ImageJ.

### Statistical analysis

Data are given as mean ± SD from three independent experiments and were compared using *t*-tests. *p* < 0.05 was deemed statistically significant.

## Results

### Triptolide reduces proliferation in melanoma cells

**Figure 1:**
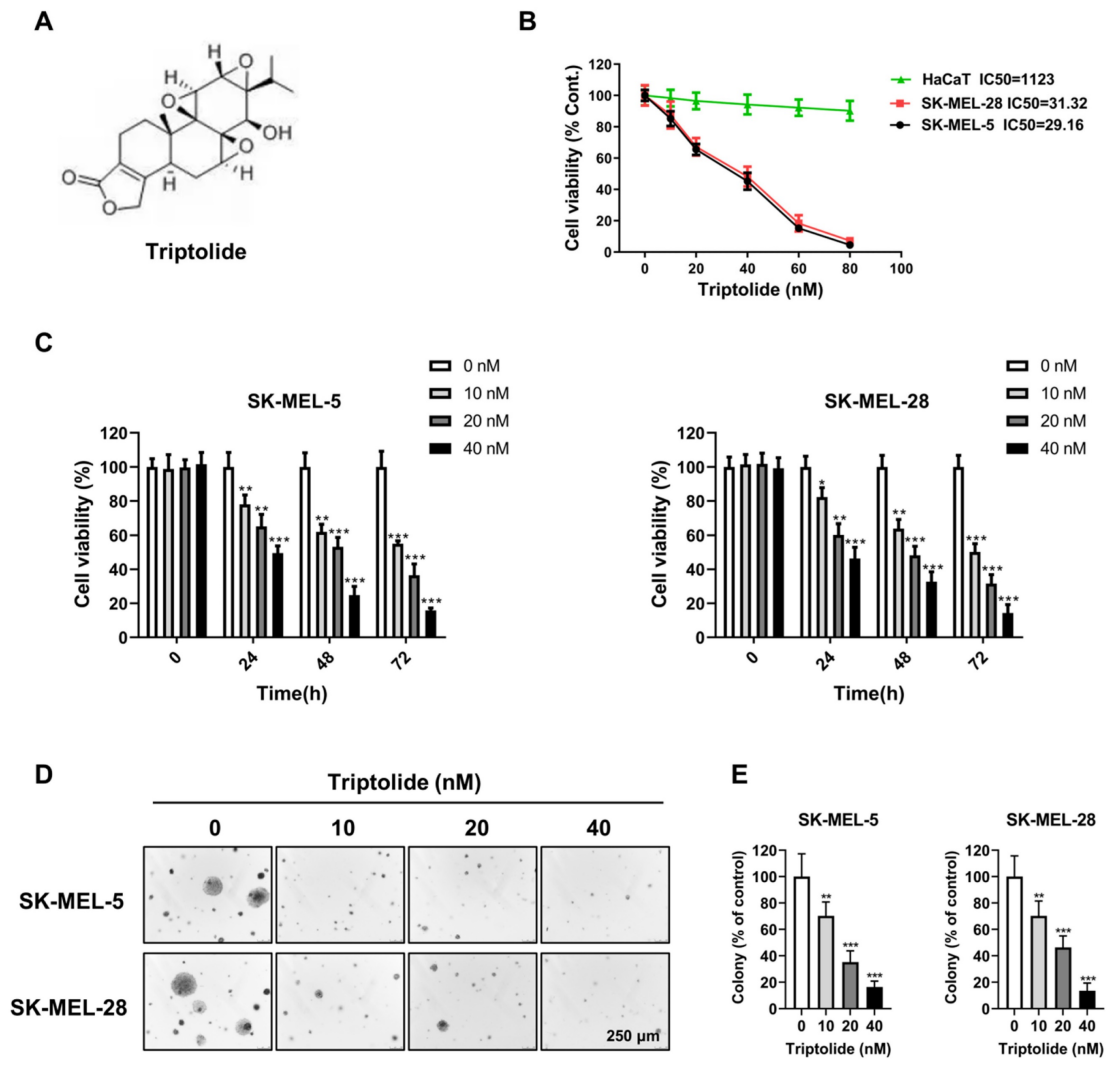
** Triptolide inhibits proliferation in melanoma cells.** Structure of triptolide. (B) Melanoma cells and human keratinocyte cell line HaCaT were exposed to varying concentrations (0, 10, 20, 40, 60, and 80 nM) of triptolide for 24 h. Viability was assessed by CCK-8 assays. (C) SK-MEL-5 and SK-MEL-28 cells were exposed to varying amounts (0, 10, 20, and 40 nM) of triptolide for 0, 24, 48, 72 and 96 h, with viability assessed by CCK-8 assays. (D) Colony formation by cells after treatment with varying concentrations of triptolide (0, 10, 20, and 40 nM) for 2 weeks. Magnification, 50×. *p<0.05; ** *p*< 0.01; *** *p*< 0.001.

Figure 1A illustrates the triptolide structure. To evaluate its effects on cell growth, SK-MEL-5 and SK-MEL-28 melanoma cells and human keratinocyte HaCaT cells were treated with varying amounts (0, 10, 20, 40, 60, and 80 nM) of triptolide for 24 h. The cell viability was markedly and dose-dependently reduced by triptolide treatment (Figure 1B). The concentrations of 0, 10, 20, and 40 nM triptolide were used for subsequent experiments. CCK-8 assays showed that triptolide dose- and time-dependently reduced melanoma cell proliferation (Figure 1C), while colony formation assays in soft agar indicated reductions in both the number and size of colonies in correspondence with the triptolide dose. Thus, triptolide can effectively inhibit the growth of melanoma cells.

### Triptolide blocks migration and invasion of melanoma cells

**Figure 2:**
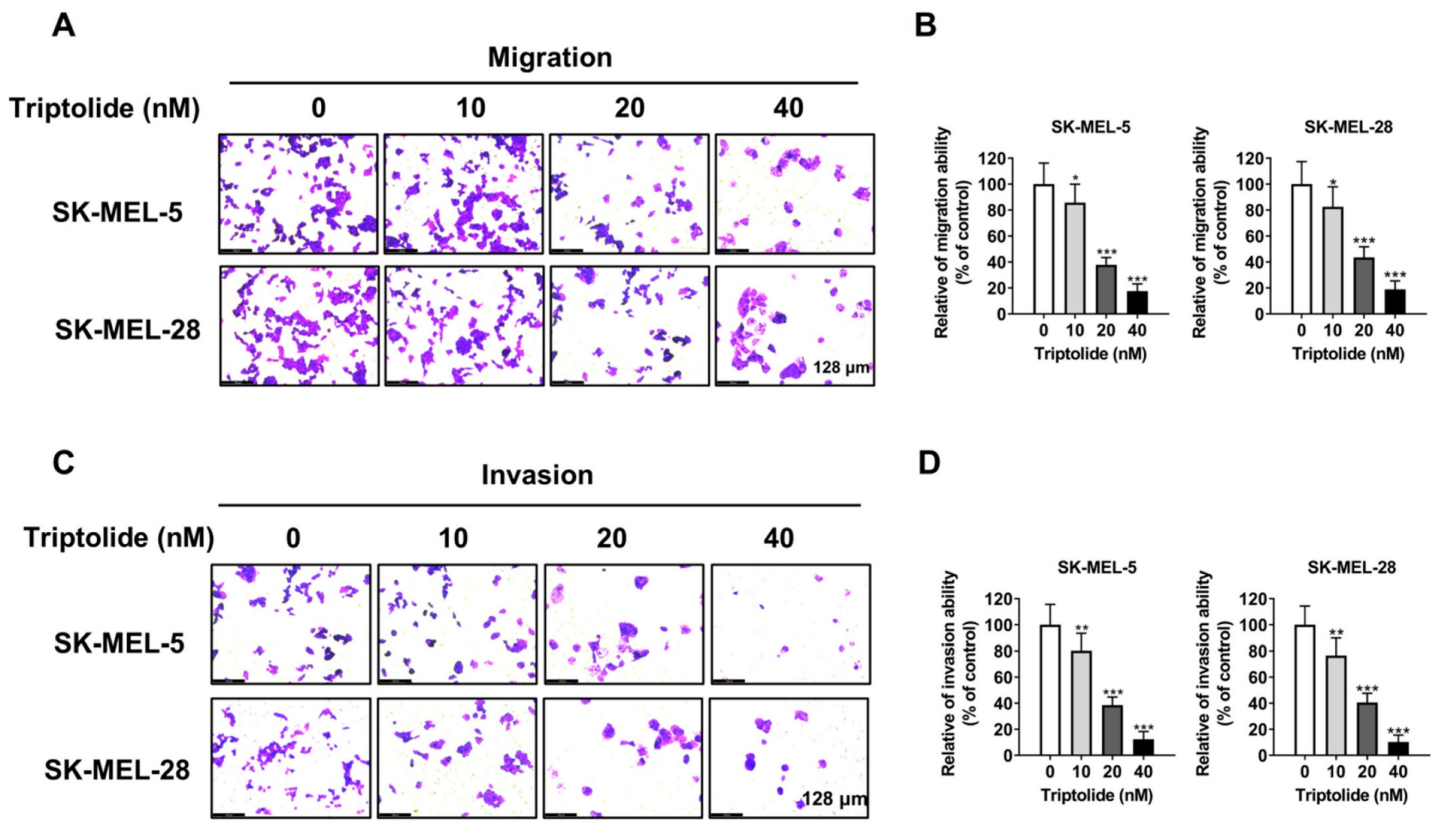
** Triptolide inhibits melanoma cell migration and invasion.** (A and B) SK-MEL-5 and SK-MEL-28 cell migration after exposure to 0, 10, 20, or 40 nM triptolide for 48 h. (C and D) Cell invasion after exposure to 0, 10, 20, or 40 nM triptolide for 48 h. *p<0.05; ** *p*< 0.01; *** *p*< 0.001.

Metastasis is strongly linked to both treatment failure and death. Transwell assays showed that triptolide markedly and dose-dependently reduced both cell migration and invasion (Figure 2A-2D), implying that it may prevent melanoma metastasis.

### Triptolide produces cell-cycle arrest in melanoma cells

**Figure 3:**
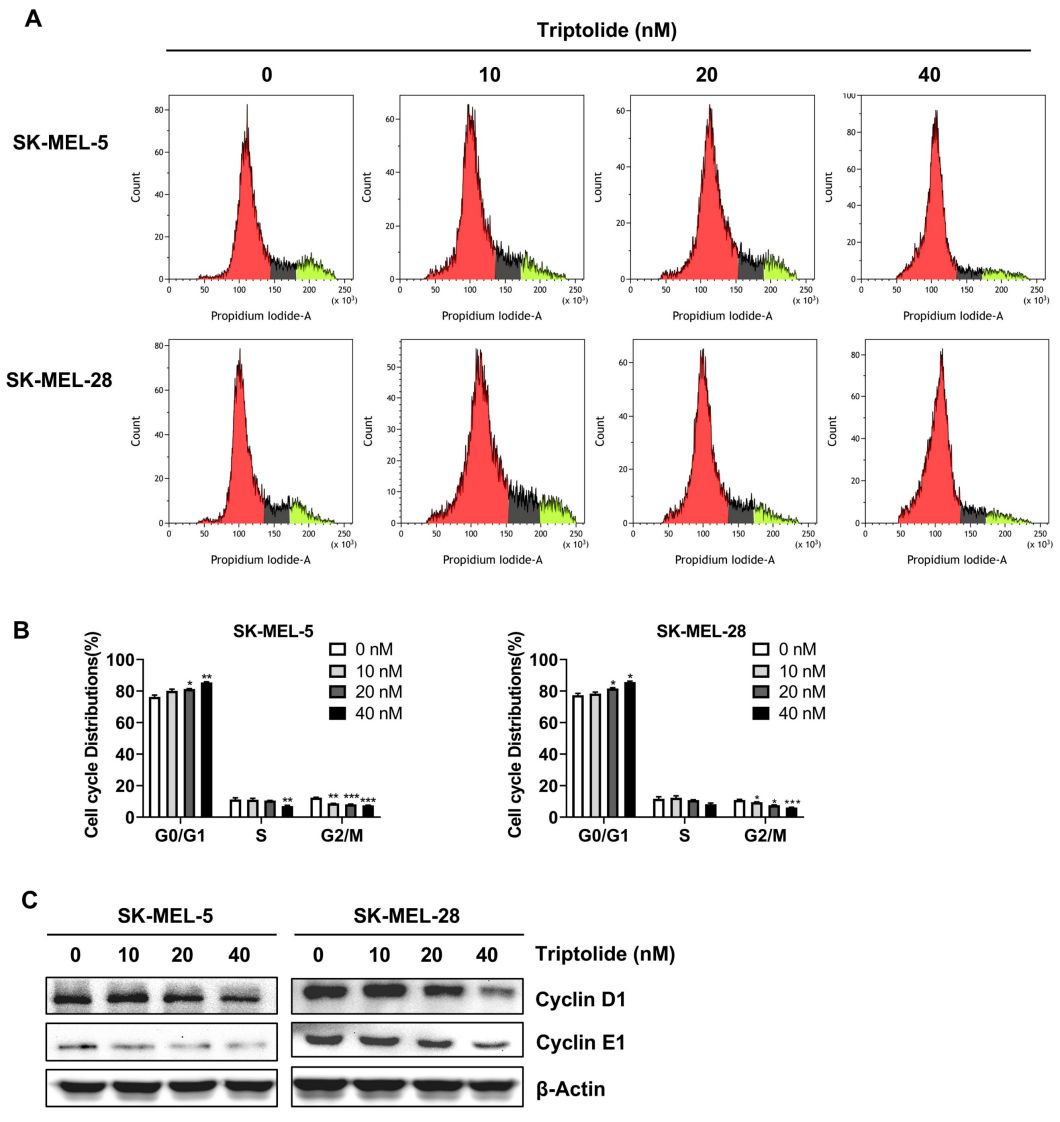
** Triptolide promotes G0/G1 cell cycle arrest in melanoma cells.** (A) Cells were exposed to 0, 10, 20, or 40 nM triptolide for 48 h before staining with PI and analysis using flow cytometry. (B) Distributions of cells in the cell cycle. (C) Protein levels of cyclins D1 and E1, shown by western blotting. *p<0.05; ** *p*< 0.01; *** *p*< 0.001.

The distributions of cells following treatment with triptolide (0, 10, 20, and 40 nM) were evaluated by flow cytometry. This demonstrated that triptolide markedly enhanced the proportions of cells in G0/G1 (Figures 3A and 3B). The impact of triptolide on G0/G1-associated proteins, namely, cyclins D1 and E1, was examined by western blotting, finding that triptolide treatment reduced the levels of both proteins in melanoma cells (Figure 3C).

### Triptolide induces melanoma cell apoptosis

**Figure 4:**
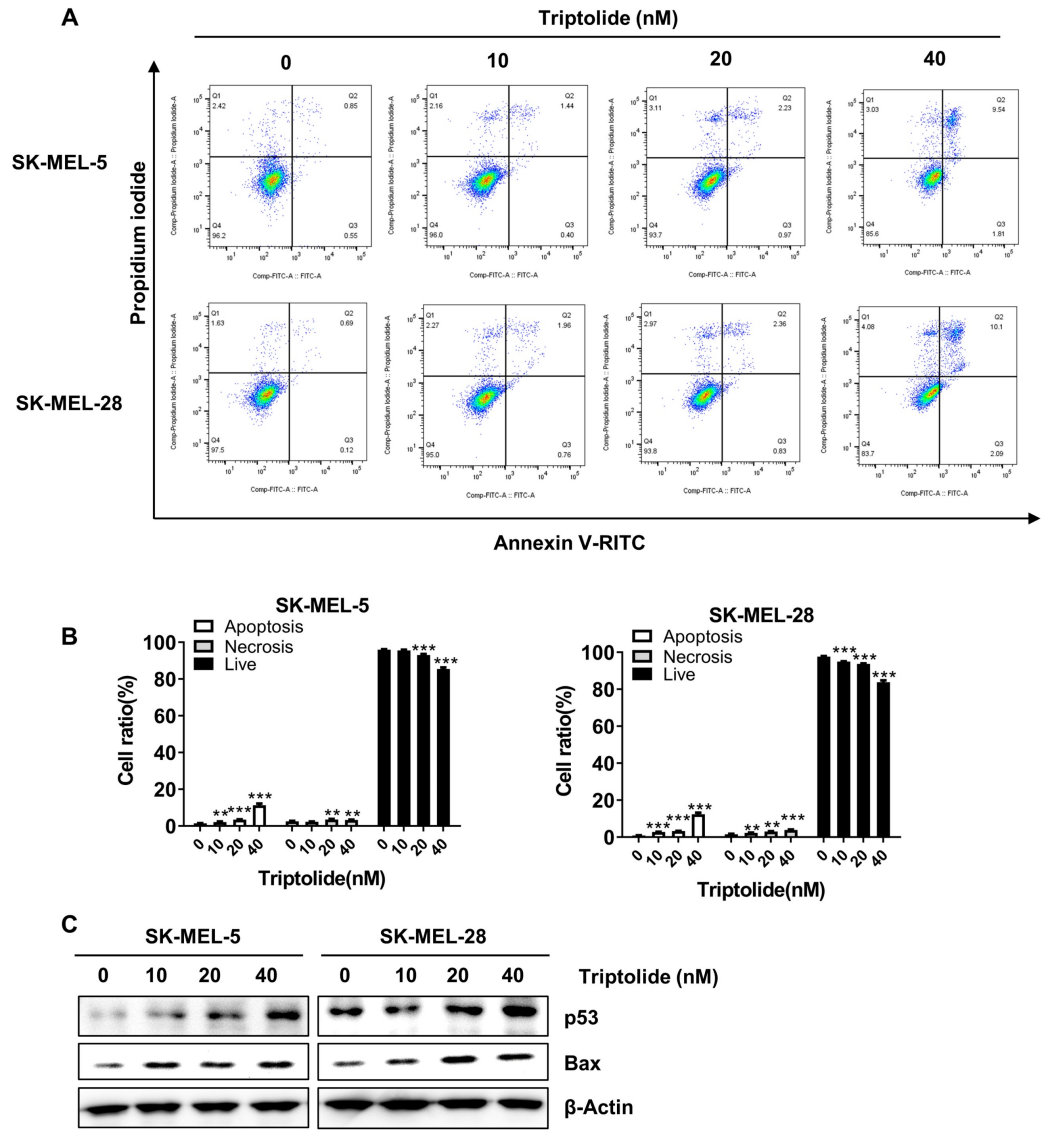
** Triptolide promotes melanoma cell apoptosis.** (A) Cells were exposed to 0, 10, 20, or 40 nM triptolide for 48 h before staining with Annexin V-FITC/PI and analysis using flow cytometry. (B) Quantification of apoptotic cell numbers following triptolide treatment. (C) Bax and p53 protein levels, shown by western blotting. *p<0.05; ** *p*< 0.01; *** *p*< 0.001.

Triptolide was found to markedly and dose-dependently increase the levels of apoptosis in melanoma cells (Figures 4A and B). Western blotting indicated markedly increased levels of the apoptosis-associated proteins p53 and Bax (Figure 4C). Thus, triptolide can induce apoptosis and thus reduce the growth of melanoma cells.

### Triptolide suppresses Src-ERK signaling in melanoma cells

**Figure 5:**
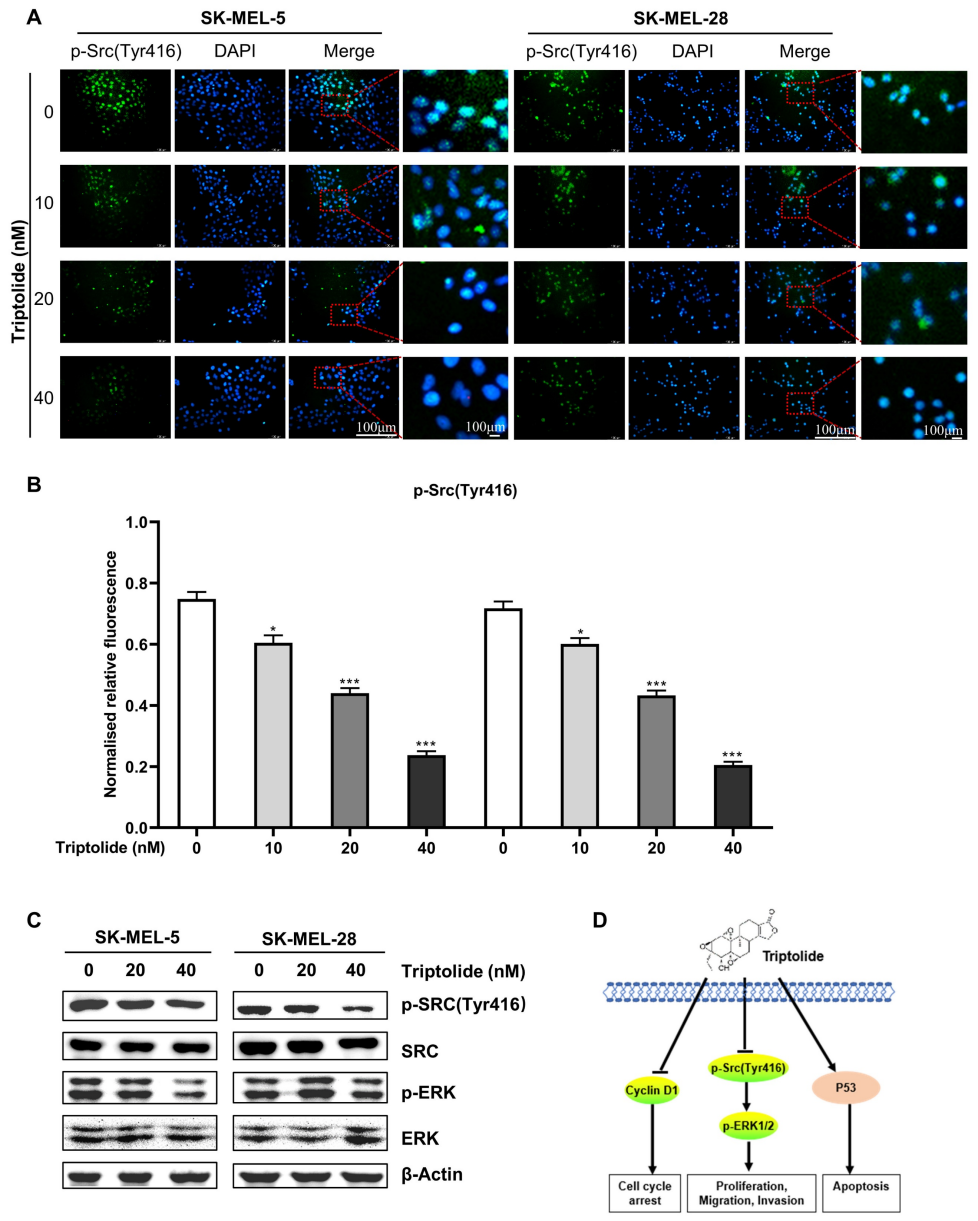
** Triptolide suppresses Src-ERK signaling in melanoma cells.** (A) and (B) Immunofluorescence staining of p-Src expression in cells after treatment with 0, 20, or 40 nM triptolide for 48 h. Scale bar, 100 μm. (C) Src, p-Src, ERK, and p-ERK protein levels, shown by western blotting. (D) Potential mechanism of triptolide inhibition of melanoma cell growth. *P < 0.05, **P < 0.01, ***P < 0.001.

As Src-ERK signaling is known to be involved in melanoma, the influence of triptolide on this axis was examined. Immunofluorescence experiments showed that triptolide significantly suppressed p-Src expression in melanoma cells (Figures 5A and B), as were the levels of p-ERK1/2 (Figure 5C). Figure 5D illustrates a proposed mechanism for the action of triptolide on melanoma cells.

### Triptolide inhibits tumor growth of cell-derived xenografts

**Figure 6:**
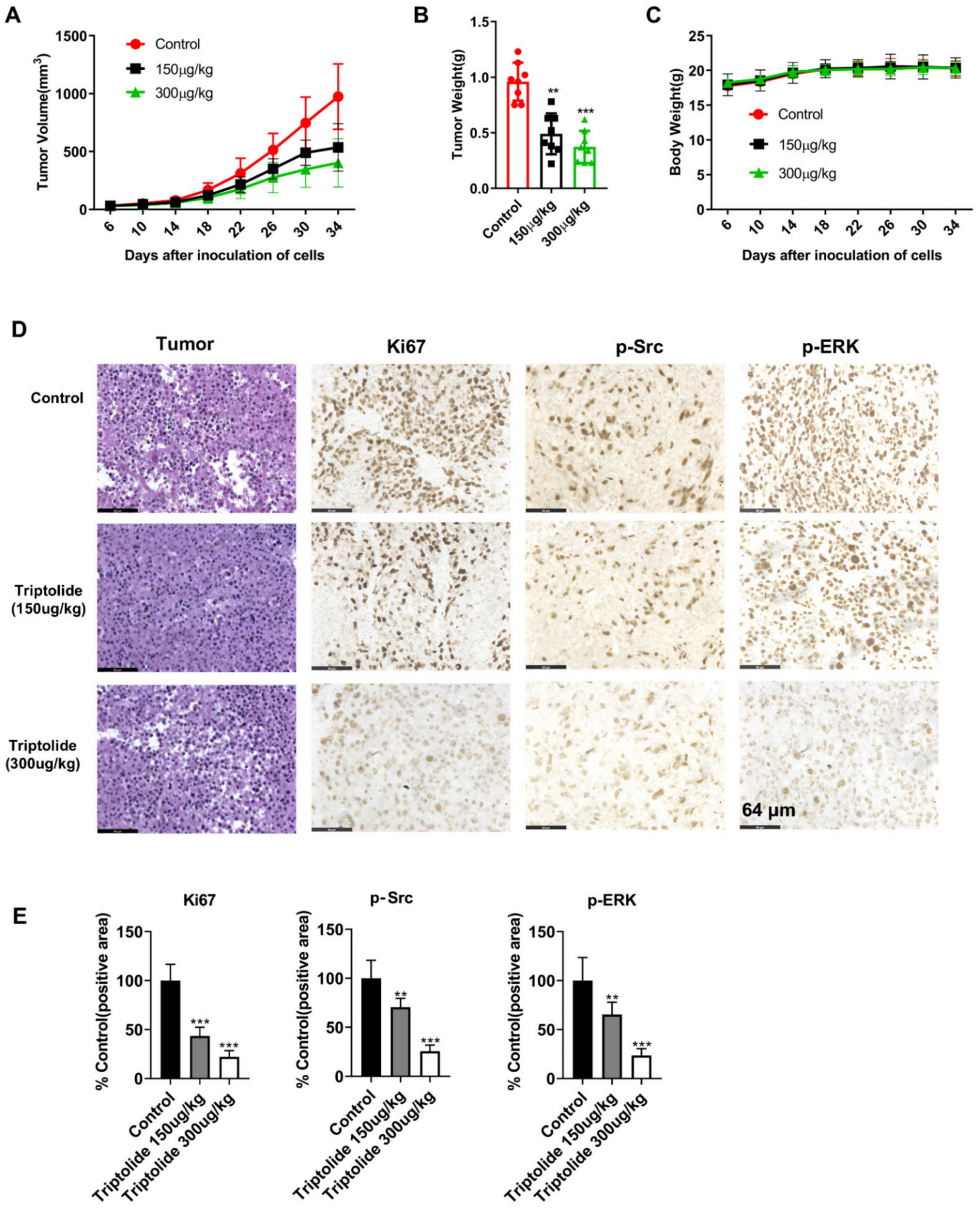
** Triptolide reduces the growth of mouse xenograft tumors.** (A) Effects of triptolide on tumor volumes over 34 days. Mice received intraperitoneal injections of vehicle or triptolide (150 or 300 μg/kg body weight), n=6 per group. Tumor volumes were recorded every four days. (B) Tumor weights at the end of the study. (C) Mouse body weights after treatment with vehicle, 150, or 300 μg/kg triptolide. (D) Immunohistochemical analysis of Ki-67, p-Src, and p-ERK in tumors; magnification, 200 x. (E) Quantification of immunohistochemical results. (F) Protein levels of p-Src and p-ERK in tumors, shown by western blotting. All data are shown as mean values ± S.D. *p<0.05; **p< 0.01; ***p< 0.001.

The action of triptolide was then determined in a mouse xenograft model produced by administration of SK-MEL-5 cells. After allowing the tumors to grow for one week, the mice were then treated with either 150 or 300 μg/kg triptolide on alternate days for four weeks. It was found that the triptolide treatment markedly reduced the growth of the tumors relative to the control group, with no significant changes in the mouse body weights (Figures 6A-C). The immunohistochemical results indicated that triptolide treatment markedly reduced Ki-67, p-Src, and p-Erk in the tumors (Figures 6D-E). These findings suggest that triptolide may be a promising treatment for melanoma.

## Discussion

Abnormal Src-ERK signaling has been observed in various physiological processes associated with cancers, including melanoma 17-22. Overexpression of SFK proteins is observed in many primary human tumors, usually the result of upstream stimulation by receptor tyrosine kinases binding to growth factors 23, 24. The Src/ERK pathway is also closely associated with tumorigenic activities, such as proliferation and metastasis, rendering it a potential target for antitumor treatment. Increased activation and phosphorylation of Src have been reported in many cancer types. A variety of phytomedicines have been observed to have antitumor activity against melanoma in both cell studies and animal models 25. Here, the chemotherapeutic effects of triptolide in human melanoma were investigated.

Triptolide is an effective anticancer constituent of *Tripterygium wilfordii* Hook.f. It has been found that* Tripterygium wilfordii* has many properties, including dehumidification, dispelling of wind, promotion of blood circulation, counteracting swelling, and pain relief, as well as detoxification and insecticidal effects. It is often used in rheumatoid arthritis, glomerulonephritis, nephrotic syndrome, and other diseases. Triptolide has antioxidant, anti-rheumatoid, anti-dementia, anticancer, and other effects 26-28. Triptolide has been shown to have potent anticancer activity in different tumor models. Mechanistically, triptolide attenuates the growth of cancer cells in association with reduced activity of STAT3 29, Notch 30, and NF-κB 30. It has also been found to reduce JAK2/STAT3 signaling and induce autophagy through the production of ROS in SKOV3/DDP ovarian tumor cells resistant to cisplatin 29, as well as suppressing the growth of lung cancer by blocking β-catenin induction of the epithelial-mesenchymal transition 31. However, its anticancer activity in melanoma has not been well studied. As melanoma is a malignancy of the skin and triptolide is effective in henoch purpura disease, we speculated that it may also be able to treat melanoma. The clinical management of melanoma typically involves chemoradiotherapy and immunotherapy, both of which are associated with numerous side effects and limited drug options. Combining multiple approaches has become the primary strategy for treating melanoma in clinical practice. Traditional cytotoxic drugs, such as dacarbazine, mortizolamide, cisplatin, etc., have shown inadequate efficacy either as monotherapy or in traditional combination regimens for melanoma treatment. Therefore, there is an urgent need for low-toxicity drugs with minimal side effects in the treatment of melanoma. Small molecule drugs derived from traditional Chinese medicine, such as triptolide, may represent a promising alternative. The experimental results confirmed this supposition, showing that triptolide could effectively block the growth of melanoma cell lines and xenograft tumors by suppressing the Src-ERK pathway.

The effects of triptolide were investigated on the growth of melanoma tumors in mice. It was found that both tumor volumes and weights, as well as the biomarker levels, were markedly reduced after triptolide treatment, relative to the controls treated with vehicle only (Figure 6). These findings suggest the potential of triptolide for treating melanoma in the future.
